# The Development and Psychometric Validation of an Arabic-Language Version of the Pain Catastrophizing Scale 

**DOI:** 10.1155/2017/1472792

**Published:** 2017-01-16

**Authors:** Huda Abu-Saad Huijer, Souha Fares, Douglas J. French

**Affiliations:** ^1^Hariri School of Nursing, American University of Beirut, Beirut, Lebanon; ^2^School of Psychology, Université de Moncton, Moncton, NB, Canada

## Abstract

*Context*. The Pain Catastrophizing Scale (PCS) is the most widely used measure of pain-specific catastrophizing.* Objectives*. The purpose of the present study was to develop and psychometrically evaluate an Arabic-language version of the PCS.* Methods*. In Study 1, 150 adult chronic nonmalignant pain patients seeking treatment at a hospital setting completed the PCS-A and a number of self-report measures assessing clinical parameters of pain, symptoms of depression, and quality of life. Study 2 employed a cold pressor pain task to examine the PCS-A in a sample of 44 healthy university students.* Results*. Exploratory factor analyses suggested a two-factor structure. Confirmatory factor analysis comparing the 2-factor model, Sullivan's original 3-factor model, and a 1-factor model based on the total score all provided adequate fit to the data. Cronbach's alpha coefficients across all models met or exceeded accepted standards of reliability. Catastrophizing was associated with higher levels of depression and increased pain intensity and interference. Catastrophizing predicted decreased quality of life, even after controlling for the contribution of gender, employment, depression, and pain interference. PCS-A scores were positively correlated with heightened experimental pain severity and decreased pain tolerance.* Conclusion*. The present results provide strong support for the psychometric properties of the PCS-A.

## 1. Introduction

Pain catastrophizing is characterized as the tendency to magnify the level of threat associated with perceived or anticipated pain, to feel helpless in the face of pain, and to focus excessively on pain sensations [1, 2]. This negative “mental set” has repeatedly been shown to be one of the most robust and reliable psychological predictors of the overall pain experience in experimental studies of pain [1–6] and in individuals undergoing acutely painful medical treatments and procedures [7–10]. Numerous studies of individuals with persistent clinical pain have also found catastrophizing to relate to a number of pain-related outcomes including pain severity, disability, activity interference, quality of life, and health care utilization [11–15]. Furthermore, treatment outcome studies have demonstrated that high levels of pain catastrophizing are associated with poor responses to pharmacological intervention [16], surgical treatment [17], physical therapies [18, 19], and psychological interventions for pain conditions [20].

As one of the most widely used measures of catastrophic thinking related to pain, the PCS has been repeatedly shown to be a valid and reliable tool for assessing pain catastrophizing. This measure is brief and may be easily integrated into existing assessment protocols with little increase in patient burden. The multidimensional nature of the PCS is also useful for tailoring clinical interventions to an individual's specific profile. It has been validated in more than 22 languages worldwide [4, 21–28] yet an Arabic-language adaptation has not been developed. The purpose of this research was to develop and psychometrically evaluate an Arabic-language version of the PCS, the PCS-A. It is hoped that the availability of an Arabic-language version will facilitate pain research and treatment in the Arab world. Two studies were conducted. Study 1 examines the factor structure of the PCS-A and the prediction of pain severity and disability in Lebanese chronic pain patients presenting for treatment in a hospital setting. Study 2 employs a cold pressor paradigm to examine acute pain responsiveness in university students from a number of Arab countries studying in Canada and compares state and trait levels of catastrophizing using the PCS-A. It is hoped that the availability of a validated Arabic-language PCS will contribute to the assessment and treatment of Arab pain patients and will be useful for pain researchers in Arab countries.

## 2. Study 1

### 2.1. Method

#### 2.1.1. Setting and Subjects

One hundred and fifty (*n* = 150) adults (aged 18 years and older) with chronic nonmalignant pain were recruited from the outpatient pain clinic and the rheumatic arthritis clinics of the AUB Medical Center. Participants reported spinal pain with or without radiculopathy and local pain syndromes of the upper and lower limbs. The research assistants (RAs) approached the patients in the waiting area, introduced themselves, and informed them about the purpose of the study. If the patient agreed to participate, the informed consent was explained and the RA secured their signatures. Afterwards, the RA administered the questionnaires in a structured interview-type format.

#### 2.1.2. Questionnaires

With the exception of the PCS-A, self-report measures have all been previously validated in Arabic with published research demonstrating psychometric properties comparable to the original English-language versions.

#### 2.1.3. Pain Catastrophizing Scale-Arabic Version (PCS-A) 

The PCS-A is an Arabic-language translation of the original 13-item PCS [1]. Like the original version, the PCS-A asks respondents to reflect on past painful experiences and to indicate the degree to which they experience the thoughts or feelings described in each item when experiencing pain; for example, “I become afraid that the pain will get worse.” Items are rated on a 5-point scale with end points of (0) “not at all” and (4) “all the time.”

#### 2.1.4. Brief Pain Inventory (BPI)—Arabic Version

The Arabic-language adaptation of the 16-item Brief Pain Inventory (BPI) assesses clinical pain severity and the degree to which pain interferes with a number of common functional domains. In addition to a bodily pain diagram, the BPI includes four pain severity items and seven pain interference items rated on 0–10 scales. Ballout et al. [29] showed that the Arabic-language translation demonstrated cultural sensitivity and adequate psychometric properties comparable to the original version when used in a sample of Lebanese cancer patients experiencing pain.

#### 2.1.5. European Organization for Research and Treatment of Cancer Quality of Life Questionnaire C30 (EORTC QLQ-C30)—Lebanese Arabic Version

The EORTC QLQ-C30 is a 30-item self-report questionnaire composed of five functional subscales (physical, role, cognitive, emotional, and social subscales), three symptom scales (fatigue, pain, nausea, and vomiting), and a global health status and quality of life scale (GHS, QoL scale). Higher scores on the functional subscales, as well as the GHS and QoL subscales, are indicative of higher levels of functioning as opposed to the symptom subscales for which higher scores are indicative of higher levels of symptom activity. The EORTC QLQ-C30 has been translated into Arabic and psychometrically validated in a large sample of Lebanese cancer patients [30].

#### 2.1.6. CES-D Depression Scale (CES-D)—Arabic Version

The Arabic CES-D scale is a 20-item measure of depressive symptoms. Symptom ratings varied from 0 to 3: 0 = rarely or none of the time and 3 = most or all of the time, 5–7 days [31]. Positive items were reverse coded and the responses were summed such that higher scores are indicative of increasing depression. The total scale score was used in the present study as recommended by Kazarian and Taher [32].

#### 2.1.7. Translation and Pilot Testing

The PCS was translated to Arabic using the forward and backward translation method. An initial professional translation was sent to two experts in the Arabic-language for evaluation. The backward translation was carried out by an independent translator with no prior knowledge of the original version. It was then validated for cultural appropriateness by four experts in the field and then pilot tested on a small sample of patients (*n* = 5) for clarity of the items, length, and the presence of distressing items. In light of culture-specific differences in spoken and written Arabic across countries, it was our goal to develop an Arabic-language version that could be used widely. As such, every effort was made to ensure the PCS-A conformed to Classic Arabic norms.

#### 2.1.8. Analysis

Statistical analyses were performed using the statistical package for the social sciences (SPSS), version 23, and STATA version 13.1 for Windows. Descriptive analyses were used to describe the characteristics of the study sample.

Exploratory Factor Analysis (EFA) was conducted to determine the factor structure of the scale. The extraction method used was principal axis factoring with oblique rotation (promax). The number of factors to be extracted was determined according to eigenvalues and the scree plot. Confirmatory factor analysis was then conducted to assess the construct validity and goodness of fit of the model revealed from the current study and to compare it to other models previously reported in the literature. The CFA goodness of fit indices used were the root mean square error of approximation (RMSEA ≤ 0.8) [33], nonnormed fit index (NNFI ≥ 0.9) [34], and comparative fit index (CFI ≥ 0.9) [30, 32].

Construct validity was also examined by correlating (Pearson's *r*) the PCS-A score with depression, pain severity, pain interference, and quality of life scores. The known-groups technique was used to compare PCS-A scores between demographic subgroups (*t*-tests). The ability of the PCS-A to predict quality of life (QoL subscale of the EORTC QLQC-30) while controlling for pain, psychosocial characteristics, and demographic variables was tested by multiple hierarchical regression analysis.

The internal consistency of the original and resulting factors was examined using Cronbach's alpha (*α*), Cronbach's alpha if item deleted, interitem correlations, and corrected item-total correlations.

### 2.2. Results

#### 2.2.1. Demographic and Clinical Characteristics

The mean participant age was 49.03 years (SD = 13.99). The majority were female (67%), completed secondary education (35%), were currently married (73%), were unemployed (55%), and had been experiencing pain for more than six months (61%). Approximately 39% of the participants had low back pain and 26% reported pain in the ankle/foot and 25.3% in the wrist/hand.

#### 2.2.2. Exploratory Factor Analysis

When examining the EFA results, only the first factor had an eigenvalue greater than one, equal to 9.69 accounting for 74.55% of the total variance. This provides strong evidence on the unidimensionality of the PCS-A. The scree plot however showed two points above “the elbow,” which led us to assess the two-factor structure later using CFA. When forced into the EFA, the two factors accounted for 80% of the total variance. Factor 1 included items 1–5 and item 12 which corresponds to the “Helplessness” subscale reported with the original scale [1]. Factor 2 included the remaining items 6–11 and 13, thus grouping the “Rumination” and “Magnification” factors in the original study and this was labelled Rumination/Magnification factor. All factor loadings were >0.4 and no items loaded on both factors (Table 1).

#### 2.2.3. Confirmatory Factor Analysis

The goodness of fit of the two-factor oblique solution suggested by EFA was assessed and compared to the one-factor model including all thirteen items also suggested by EFA and the oblique three-factor model proposed by Sullivan et al. [1]. The two-factor model showed good fit with CFI > 0.9 and NNFI > 0.9 while RMSEA = 0.11 > 0.08 which still indicates an acceptable fit. The fit of the original three-factor model was also acceptable and identical to the two-factor model. The one-factor model also showed adequate fit but was slightly poorer than the other two. The CFA indices are summarized in Table 2.

#### 2.2.4. Reliability

Within the two-factor model, values of Cronbach's alpha were well above the standard of reliability with a coefficient of 0.97 for the total score, 0.96 for factor 1, and 0.95 for factor 2. These values are all comparable to or higher than values obtained for three subscales when created according to Sullivan's item groupings. All corrected item-total correlations were high ranging from 0.76 to 0.90 for the one-factor model and from 0.82 to 0.90 for the Rumination/Magnification dimension in the two-factor model and from 0.76 to 0.90 for the Helplessness dimension.

#### 2.2.5. Concurrent and Predictive Validity

PCS-A scores were significantly higher in females (M = 28.6, SD = 14.3) compared to males (M = 20.2 SD = 12.8; *t*(141) = −3.44, *p* = 0.001). PCS-A scores were positively correlated with depression scores (*r* = 0.55, *p* < 0.001) and negatively correlated with quality of life scores (*r* = −0.45, *p* < 0.001), physical functioning (*r* = −0.46, *p* < 0.001), emotional functioning (*r* = −0.55, *p* < 0.001), and role functioning (*r* = −0.47, *p* < 0.001) scores. The correlations with pain interference and pain severity were also significant (*r* = 0.37, *p* < 0.001 and *r* = 0.26, *p* = 0.002).

The predictive validity of the PCS-A was assessed using multiple hierarchical linear regression (Table 3). Variables significantly associated with quality of life at the univariate level were entered as follows: in the first step, demographic characteristics were entered; in the next step pain and psychosocial variables were entered followed by PCS-A score in the last step. Quality of life was predicted by PCS-A scores even after controlling for gender, employment, depression, and pain interference (*B* = −0.36, 95% CI from −0.64 to −0.09, and *p* value < 0.05).

## 3. Study 2

### 3.1. Participants

A sample of forty-four (*n* = 44; 13 females, 31 males) Arab-speaking international students attending a French-Canadian university were recruited by posters placed on campus billboards and via mass email recruitment drives. Participants were young adults (mean age in years = 24.2, SD = 5.7) from Morocco (50%), Tunisia (38.6%), Algeria (9.1%), and Lebanon (2.3%).

### 3.2. Measures and Apparatus

#### 3.2.1. Pain Catastrophizing Scale-Arabic Version (PCS-A)

The PCS-A represented the Arabic-language translation of the original PCS [1]. Like the original version, the PCS-A consisted of 13 items describing different thoughts and feelings that individuals may experience when they are in pain. The PCS-A and a modified version which asks participants to reflect on thoughts and feelings they experienced during the experimental pain task (state catastrophizing) were employed.

#### 3.2.2. Spielberger State Anxiety Inventory (STAI)

The STAI consists of two 20-item subscales comprised of 20 short statements reflecting cognitive symptoms of anxiety. Participants are asked to rate how they feel either at the moment (State Anxiety) or in general (Trait Anxiety) on a four-point scale, from 1 (not at all) to 4 (very much so). This scale is widely used as a research tool and has demonstrated acceptable internal consistency, test-retest reliability, and construct validity [35].

#### 3.2.3. Numeric Rating Scale (NRS)

This scale comprises a vertical line graphic labelled with intensity-denoting adjectives and numbers ranging from 0 (no pain) to 100 (maximum tolerable pain). These rating scales are widely used measures of subjective pain and have been shown to possess adequate reliability and validity [36].

#### 3.2.4. Cold Pressor (Wine Well Chiller Co., Inc.)

The Model II-BX consists of a refrigerated tank with a 30 cm diameter opening that constantly circulates and maintains 40 liters of water at an average temperature of 2 ± 0.5°C.

### 3.3. Method

Participants began by completing the PCS-A and the STAI. The 0–100 numeric rating scale (NRS) was explained to participants prior to the cold pressor test which proceeded in the following manner: participants were instructed to immerse their nondominant hand into the circulating ice water and to indicate when they first began to experience pain (threshold). They were instructed to leave their hand immersed until they could no longer tolerate the pain, at which point they could remove their hand (tolerance). Time of immersion in seconds was calculated using a stopwatch. Participants then provided an NRS rating of the overall level of pain associated with the immersion task and they completed the state version of the PCS-A.

### 3.4. Results

Visual inspection of study data and formal measures of skewness and kurtosis revealed that the distribution of scores on all variables did not deviate significantly from normality.

Reliability was assessed using Cronbach's alpha and interitem correlations. A high degree of internal consistency was found within both the original trait version and the modified state version of the PCS-A whether examined using the 13-item total score (range of *α* = 0.88–0.92; range of interitem *r* = .57–.65) or within each the three original subscales (range of *α* = 0.63–0.89; range of interitem *r* = 0.45–0.71).

As shown in Table 4, males and females did not differ significantly on PCS for both state and trait catastrophizing. Males and females did differ however on cold pressor performance with females reporting significantly lower pain threshold and tolerance times compared to their male counterparts. No significant sex differences in NRS values of overall pain severity were observed.

Table 5 presents the correlations among study variables. Consistent with previous studies, state catastrophizing (thoughts and feelings experienced during the pain task) assessed proximal to pain induction, demonstrated a stronger and a more consistent relationship across pain measures compared to ratings of participants' tendency to catastrophize in response to pain generally. Higher PCS-A scores were associated with heightened levels of experimental cold pressor pain, as suggested by lower pain thresholds, higher pain intensity ratings, and decreased pain tolerance.

## 4. Discussion and Conclusion

### 4.1. Factor Structure of the PCS-A

Previous studies of the validity and reliability of the PCS have yielded results that were psychometrically comparable to Sullivan's original 3-factor structure: Rumination, Magnification, and Helplessness. This factor structure, however, was not invariant across investigations. Some of the studies suggested a 3-factor solution with similar item groupings [22, 28] while another suggested a 3-factor structure but with different item groupings [21]. The present data yielded a clear 2-factor structure with factor I reproducing the original Helplessness subscale [1] and factor II a combination of Magnification and Rumination. The item-loadings are different again from the results of Osman et al. [37] that previously suggested 2 factors.

Regarding the CFI results, our findings are similar to a number of previous factor analytic studies of the PCS [21, 38–40] and accounted for a greater percentage of variance compared to the others [33, 34]. Our NNFI findings are similar to Goubert et al. [34] and higher than results reported by Huijer et al. [30] obtained in the context of the validation of another Arabic-language self-report measure. The RSMEA findings are similar to Huijer et al. [30] and Goubert et al. [34] but higher than other studies [21, 38, 39].

The factor structure of the PCS, whether assessed using exploratory or confirmatory FA approaches, has varied across studies. In the present study, the failure to replicate factor separation of the original Magnification and Rumination factors is not surprising given the known variation across investigations. It is important to note that the two-factor model obtained in the present study, Sullivan's original 3-factor model, and the single-factor model based on the total score all provided very good fit to the data. As such, users of the PCS-A should feel comfortable scoring this measure according to Sullivan's original 3-factor item groupings and when using the total score, which is used most frequently in studies involving the PCS.

### 4.2. Reliability

Cronbach's alpha values in the present study were comparable to or exceeded values reported in previous studies [1, 21, 22, 37, 38] suggesting a very high level of internal consistency. A similar pattern of findings was observed when examined in pain-free young adults. Taken together, these results suggest the PCS-A is reliable and compares well to the original version.

### 4.3. Validity

In Study 1, higher scores on catastrophizing were associated with higher levels of clinical pain severity and a greater degree of pain-related interference. Catastrophizing also showed a positive relationship to self-reported symptoms of depression and anxiety. This is consistent with previous studies showing a strong correlation between PCS, pain intensity, pain interference, depression, disability, emotional functioning, anxiety, physical functioning, and general health [21, 25, 41]. Regression analyses revealed that patient's responses on the PCS-A predicted decreases in general quality of life even after accounting for the significant contribution of demographic factors, pain severity, and depression.

In Study 2, increasing PCS-A scores were associated with heightened levels of experimental cold pressor pain, as suggested by lower pain thresholds, higher pain intensity ratings, and decreased pain tolerance. Furthermore, the pattern of the present findings is consistent with previous research suggesting that state catastrophizing (situation-specific thoughts) shows stronger and more consistent relationships with both clinical and experimental pain responsiveness compared to general measures of catastrophizing which rely on memory of past painful events [5, 42]. Study 2 findings also suggest that the linguistic reach of the PCS-A extends beyond Lebanon and may be used to good effect with individuals in other Arab countries.

Overall, the present results provide strong support for the psychometric properties of the Arabic-language adaptation of the PCS-A. Although further research is needed to replicate and expand the current psychometric investigation, particularly among clinical pain groups in Arab-speaking countries outside of Lebanon, it is with confidence that we recommend the PCS-A for use by researchers and clinicians alike.

## Figures and Tables

**Table 1:** EFA using principle axis factoring and promax rotation.

Items	Factors	
	1	2
I worry all the time about whether the pain will end	0.09	**0.73**
I feel I can't go on	0.11	**0.77**
It's terrible and I think it's never going to get any better	0.22	**0.73**
It's awful and I feel that it overwhelms me	0.16	**0.77**
I feel I can't stand it anymore	−0.04	**0.98**
I become afraid that the pain will get worse	**0.73**	0.15
I keep thinking of other painful events	**0.92**	−0.10
I anxiously want the pain to go away	**0.71**	0.15
I can't seem to keep it out of my mind	**0.78**	0.15
I keep thinking about how much it hurts	**0.68**	0.28
I keep thinking about how badly I want the pain to stop	**0.72**	0.23
There's nothing I can do to reduce the intensity of the pain	0.35	**0.48**
I wonder whether something serious may happen	**0.66**	0.22

**Table 2:** Goodness of fit indices of the different used models.

		RSMEA	CFI	NNFI
Model 1	One factor (13 items)	0.15	0.90	0.88
Model 2	Two oblique factors (7 + 6 items)	0.11	0.95	0.94
Model 3	Three oblique factors (6 + 3 + 4 items)	0.11	0.95	0.94

Model 2 revealed by the current study and Model 3 suggested by Sullivan et al. [1].

**Table 3:** Summary of hierarchical regression analysis for PCS-A predicting quality of life (QoL).

Variable	Step 1			Step 2			Step 3		
	*B*	SE(*B*)	CI	*B*	SE(*B*)	CI	*B*	SE(*B*)	CI
Gender	−1.42	4.65	(−10.62; 7.78)	2.05	4.04	(−5.95; 10.05)	3.45	3.98	(−4.43; 11.33)
Employment	11.09^*∗*^	4.34	(2.49; 19.69)	8.12^*∗*^	4.03	(0.14; 16.10)	7.30	3.95	(−0.52; 15.12)
CES-D				−0.41^*∗*^	0.20	(−0.80; −0.01)	−0.21	0.21	(−0.62; 0.20)
Pain interference				−3.18^*∗*^	0.89	(−4.94; −1.41)	−3.01^*∗*^	0.88	(−4.74; −1.28)
PCS-A							−0.36^*∗*^	0.14	(−0.64; −0.09)
Adjusted *R*^2^	0.05			0.32			0.36		
Δ*R*^2^	0.07^*∗*^			0.25^*∗*^			0.04^*∗*^		

^*∗*^*p* < 0.05.

**Table 4:** Means and standard deviations for study variables.

	Total sample		Males		Females		*t*
	M	SD	M	SD	M	SD	
PCS-A	21.9	9.5	22.16	8.27	21.23	12.37	−0.25
PCS-A state	18.9	11.8	17.97	10.89	21.08	14.06	0.79
STAI (*n* = 19)							
Trait	39.8	10.5	37.15	7.98	45.67	7.98	1.73
State	31.6	7.6	29.69	7.55	35.67	9.07	1.68
Pain							
Threshold	19.2	12.9	22.10	14.09	13.15	7.13	−2.67^*∗∗*^
Tolerance	99.3	88.2	113.20	94.15	66.09	63.18	−1.93^*∗∗*^
NRS pain	71.9	18.5	71.23	18.03	73.46	20.14	0.36

^*∗∗*^*p* < 0.01.

**Table 5:** Intercorrelations among study variables.

Scales	(1)	(2)	(3)	(4)	(5)	(6)	(7)
(1) PCS-A	—	0,54^*∗∗*^	0,15	0,12	−0,09	−0,30^*∗*^	0,13
(2) PCS-A state		—	−0,12	−0,11	−0,37^*∗∗*^	−0,53^*∗*^	0,53^*∗∗*^
(3) STAI trait			—	0,55^*∗∗*^	−0,10	−0,09	−0,15
(4) STAI state				—	0,15	−0,09	−0,27
(5) Threshold					—	0,52^*∗∗*^	−0,38^*∗∗*^
(6) Tolerance						—	−0,40^*∗∗*^
(7) NRS Pain							—

^*∗*^*p* < 0.05; ^*∗∗*^*p* < 0.01.
